# Knowledge and Attitudes of Parents of School-Aged Children Regarding Vaccinations, and an Analysis of Measles and Pertussis Vaccination Coverage Using the Example of the City of Radomsko in Central Poland

**DOI:** 10.3390/vaccines13080869

**Published:** 2025-08-16

**Authors:** Paweł Nowicki, Magdalena Górajska, Anna Garus-Pakowska

**Affiliations:** 1 Department of Nutrition and Epidemiology, Medical University of Łódź, 90-419 Łódź, Poland; pawel.nowicki@stud.umed.lodz.pl; 2 Centre of Mathematics and Physics, Łódź University of Technology, 93-590 Łódź, Poland; magdalena.gorajska@p.lodz.pl

**Keywords:** vaccination coverage, vaccination knowledge, parental attitudes, vaccine hesitancy, VAX scale, immunization programs, measles, pertussis, public health, education, Poland

## Abstract

**Background**: Vaccinations are crucial for preventing infectious diseases. Parental knowledge and attitudes significantly impact vaccination decisions. **Methods**: This study analyzed parental knowledge and opinions on childhood vaccinations (focus: measles, pertussis) and assessed vaccination coverage rates in Radomsko, Poland. A cross-sectional study (Jan–Mar 2025) combined the following: (1) parent questionnaires (children aged 6–11 years), including opinions based on the validated VAX scale and (2) analysis of official vaccination coverage data (sanitary inspection). Statistical analysis included descriptive statistics and logistic regression; results are presented as odds ratios (OR). **Results**: A total of 459 parents participated (mean age 38.9 years, 95% female, 67% Master’s-level education). **Conclusions**: Most correctly identified measles (92%) and pertussis (85%) vaccines as mandatory. Considerable confusion existed about newer mandatory vaccines and varicella (78% incorrectly thought mandatory). Analysis revealed the influence of both knowledge and opinions from the VAX scale on vaccination decisions. Higher parental education significantly increased vaccination adherence for pertussis (OR = 2.03; *p* < 0.001) and both diseases (OR = 1.83; *p* < 0.001). While general vaccination awareness was high (97%), detailed knowledge of Poland’s mandatory schedule was alarmingly low, especially for newer vaccines. Parental education level is a key determinant of both accurate knowledge and vaccination compliance. Targeted educational interventions are urgently needed to improve parental understanding and support public health goals.

## 1. Introduction

Vaccinations, as a form of primary prevention, constitute the cornerstone of societal protection against infectious diseases. They represent the most effective public health intervention for combating communicable diseases. The eradication of smallpox in 1980 [1] and the reduction in infections from other infectious diseases serve as evidence of the efficacy of vaccination programs. A monumental achievement of vaccination is the annual protection of approximately 2–3 million children, which has contributed significantly to the global decline in the mortality rate due to infectious diseases—from 65 per 1000 live births in 1990 to 29 per 1000 live births in 2018 [2]. Vaccines not only provide effective protection against diseases but also mitigate the severity of infections and reduce the risk of serious complications. Consequently, this leads to fewer hospitalizations and lowers associated healthcare costs.

Globally, public health indicators (e.g., incidence rates, mortality rates, etc.) are systematically monitored. Approaches to developing national health policy vary across countries, reflecting local epidemiological contexts. Within this framework, each state establishes immunization programs aligned with its current national and regional epidemiological situation. The World Health Organization (WHO) monitors global health threats, supports countries in health policy planning, and defines pathogen control strategies [3].

### 1.1. Immunization Programs in Other Countries

In the European Union (EU), vaccines are approved based on common indications; however, individual member states differ in their immunization policies [4]. A key distinction highlighted by anti-vaccination movements is the mandatory nature of vaccinations. In Scandinavian countries—where public health literacy is notably high—compulsory vaccination is deemed unnecessary. These nations (Sweden, Norway, and Finland) maintain exceptionally high vaccination coverage rates. Conversely, in Romania, the absence of mandates has led to declining immunization rates, resulting in a measles epidemic ongoing since 2023 [5].

In response to shifting public health indicators across the European Region, many countries are tightening vaccination requirements. France exemplifies this trend, expanding its mandatory childhood vaccines from 3 to 11 in January 2018. Current compulsory immunizations in France target diphtheria, tetanus, poliomyelitis, pertussis, Haemophilus influenzae type b (Hib), hepatitis B, pneumococcal disease, measles, mumps, rubella, and meningococcal serogroup C.

Germany strengthened regulations in 2017, authorizing kindergarten and school administrators to deny admission to unvaccinated children. Parents refusing vaccination face fines of up to EUR 2500 [6]. Similarly, Italy requires childhood immunization for preschool enrollment [7].

In Canada and the United States, unvaccinated children face comparable educational access restrictions. Australia integrates immunization with social policy through its “No Jab, No Pay” initiative (effective 2016), which bars unvaccinated children from educational institutions and withholds childcare subsidies [8]. This policy has substantially increased Australia’s herd immunity, with vaccination rates rising annually.

New Zealand exemplifies an alternative approach; though vaccinations are non-mandatory, intensive socio-educational campaigns enhance population health awareness.

### 1.2. Vaccination Surveillance in Poland

In Poland, vaccination oversight is administered by the Chief Sanitary Inspector (Polish: Główny Inspektor Sanitarny, GIS). Based on the country’s epidemiological situation, recommendations are established for both mandatory and recommended immunizations. Mandatory vaccinations in Poland are provided free of charge, while recommended vaccines are categorized as either publicly funded or paid.

The first Immunization Program (Polish: Program Szczepień Ochronnych, PSO), introduced in the 1960s, included vaccinations against smallpox, tuberculosis, diphtheria, tetanus, and pertussis. Over the past 60+ years, the program has undergone multiple revisions in response to evolving epidemiological needs and medical advances, including the introduction of new vaccines.

The 2025 Immunization Program in Poland encompasses mandatory vaccinations against 12 infectious diseases and 6 recommended immunizations (Table 1).

**Figure 1:**
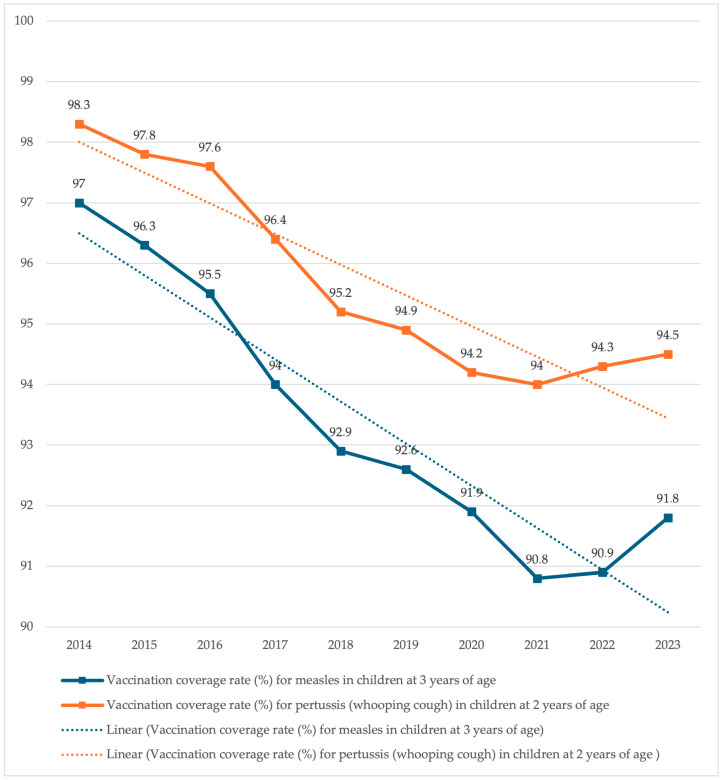
Changes in measles and pertussis vaccination rates (2014–2023) in Poland [12,13,14,15,16,17,18,19,20,21].

Analysis of measles and pertussis vaccination coverage data from 2014 to 2023 revealed a pronounced downward trajectory. In the initial observation period, vaccination coverage remained high, exceeding 95% for both diseases (recommended in the event of a population threat [10,11]). However, subsequent years exhibited a systematic reduction in the proportion of vaccinated children, potentially compromising epidemiological stability at national and regional levels. This declining trend is visually represented in Figure 1.

### 1.3. Characteristics of Pertussis and Measles

Pertussis (whooping cough) is a disease caused by the bacteria *Bordetella pertussis* and *Bordetella parapertussis*, occurring worldwide. It spreads via droplet transmission. Its infectiousness is high [22]. The only reservoir for pertussis is humans. It is characterized by paroxysmal, exhausting coughing that often leads to dyspnea and/or vomiting.

The course of pertussis is divided into three stages:

1. Catarrhal stage: Resembles a common cold (rhinitis, low-grade fever, and mild cough). This phase lasts approximately 1–2 weeks and is the most infectious.
2. Paroxysmal stage: Features episodes of suffocating cough ending with “wheezing” breathing.
3. Convalescent stage.

**Figure 2:**
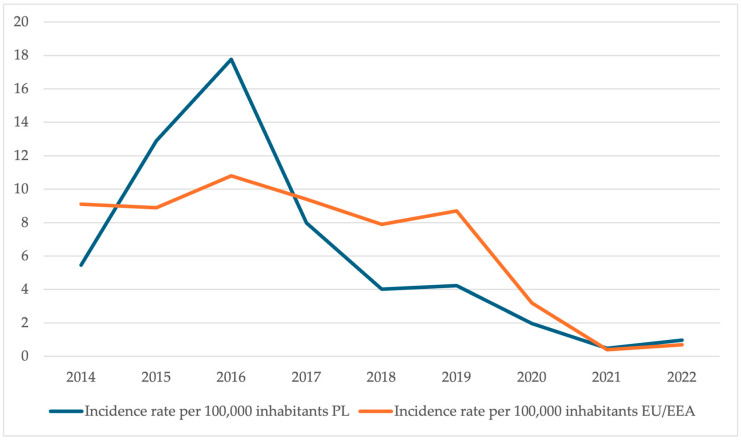
Pertussis incidence rates in Poland [12,13,14,15,16,17,18,19,20,21] compared to EU/EEA averages [24,25,26,27,28,29,30] in 2014–2022. * Data excluding the United Kingdom as of 2020.

Complications of pertussis include pneumonia, atelectasis, pneumothorax, seizures, central nervous system (CNS) hemorrhages, and hypoxic encephalopathy. The group most at risk for pertussis complications are young children and infants under 6 months of age [23]. From 2014 to 2022, pertussis incidence in Poland fluctuated, peaking in 2016 at 17.77 cases per 100,000 population. This significantly exceeded the average incidence for EU/EEA countries (European Union/European Economic Area) (10.8 cases per 100,000 population) (Figure 2).

According to epidemiological reports, in Poland in 2023, there were 922 pertussis infections (incidence 2.45) [31], during the same period in the WHO European Region, 87,000 cases were recorded—the highest in 10 years [32]. A sharp increase in pertussis infections was registered in Poland in 2024; there were 32,430 of them (incidence 86.33) [31].

Measles is an acute febrile viral disease, highly contagious. It is caused by the measles virus, belonging to the *Paramyxoviridae* family and *Morbillivirus* genus. The source of infection is humans; an infected person is infectious for about 8 days (3–5 days before the rash and during the first 3 days of the rash occurring) [33]. It spreads via droplet route. A characteristic symptom is Koplik’s spots on the buccal mucosa. Initial symptoms resemble a common cold (high fever, cough, runny nose, and conjunctivitis). The most common complications are pneumonia, bronchitis, otitis media, and subglottic laryngitis [34]. One of the most serious complications of measles is subacute sclerosing panencephalitis (SSPE—subacute sclerosing panencephalitis), which may manifest only several years after contracting measles [35].

**Figure 3:**
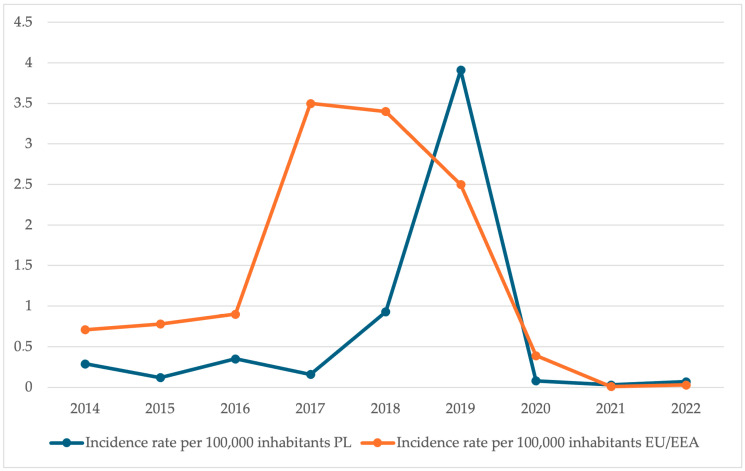
Measles incidence rates in Poland [12,13,14,15,16,17,18,19,20,21] compared to EU/EEA averages [36,37,38] in 2014–2022.

In the period 2014–2017, the average measles incidence in the EU/EEA systematically increased, reaching 3.5 cases per 100,000 inhabitants in 2017. During the same time, the measles incidence rate in Poland was 0.16 cases per 100,000 inhabitants. The turning point in the epidemiological situation in Poland was the year 2019, when measles incidence sharply increased (3.91/100,000), thereby exceeding the EU/EEA average (2.5/100,000). The sharp decline in 2020 can be linked to actions undertaken during the COVID-19 pandemic (Figure 3).

In 2023, there were 35 cases of measles in Poland (incidence 0.09); in 2024, there were 279 cases of measles (incidence 0.74) [31]. The WHO European Region recorded 58,000 cases of measles in 2023 [32].

The aim of the study was to analyze the knowledge and opinions of parents of children aged 6–11 years regarding protective vaccinations, with particular emphasis on vaccination against pertussis and measles, as well as an analysis of data concerning the vaccination rate against these diseases among children born between 2014 and 2019.

## 2. Materials and Methods

### 2.1. Study Design

The study was conducted between January and March 2025 in the city of Radomsko (Central Poland).

Data were collected in two stages: firstly, by gathering parents’ opinions on protective vaccinations using a questionnaire and secondly, by analyzing statistical data on the implementation of measles and pertussis vaccinations among children born between 2014 and 2019, obtained from the vaccination surveillance system maintained by the State Sanitary Inspectorate in Radomsko.

In the instructions accompanying the questionnaire (Supplementary Materials), respondents were informed of the study’s purpose, anonymity, and the voluntary nature of participation. Permission to conduct the study was obtained from the Head of the Department of Education and Culture of the Radomsko City Office, the principals of the respective primary schools, and the State District Sanitary Inspector in Radomsko.

The study project received a positive opinion from the Bioethics Committee of the Medical University of Lodz on 14 January 2025, Ref. No. RNN/40/25/KE/KE.

### 2.2. Study Setting

The study setting was Radomsko—a mid-sized city in Central Poland, with a population of 42,233 (as of 3 January 2025). Demographically, women constitute 53.1% of residents compared to 46.9% men. There are 10 public primary schools in Radomsko, attended by 3428 children (as of 3 January 2025). Familial structure data indicates that 70.5% of the city’s 13,116 households have children, while adult education levels show that 24.3% of households hold higher education degrees, while 34.2% have completed secondary education. Economically, the median gross monthly salary is PLN 6655.76 (≈EUR 1530.00) by residence and PLN 6212.00 (≈EUR 1428.00) by workplace [39,40].

### 2.3. Cross-Sectional Survey on Parental Knowledge and Opinions

The study participants were parents of children attending grades 1–3 at all public primary schools in Radomsko. All 10 public primary schools in the city were invited to participate. During the 2024/2025 school year, these grades were attended by 1266 children. Ultimately, 8 schools confirmed their willingness to participate in the study, involving 1144 children enrolled in grades 1–3.

Electronic questionnaires with completion instructions were distributed to respondents via the electronic school register system by school principals. Additionally, posters encouraging participation in the study (Supplementary Materials) were displayed in schools, and a QR code was generated to facilitate access to the research instrument.

The study involved 459 participants, with a questionnaire response rate of 40.12%. No formal sample size calculation was performed, as the study aimed for a complete census by inviting all public schools in Radomsko to participate. The final sample reflects the number of respondents who voluntarily agreed to take part.

### 2.4. Research Instrument

At the beginning of the questionnaire, respondents were asked to provide consent to participate in the study and to confirm that they had a child in grades 1–3 of primary school. This ensured that only individuals who were parents of school-aged children and had consented to participate were included in the study.

The questionnaire consisted of five sections:

- Questions on the socio-demographic characteristics of respondents
- Questions assessing knowledge about vaccinations.
- Parental practices regarding child vaccinations.
- Assessment of access to vaccination-related information.
- The validated ‘Vaccination Attitudes Examination (VAX) Scale’, evaluating respondents’ attitudes towards protective vaccinations. The translation into Polish was performed by Borchet, Iwanowska (Institute of Psychology, Faculty of Social Sciences, University of Gdańsk), Bałandynowicz-Panfil (Department of Sustainable Market Processes, Faculty of Economics, University of Gdańsk), and Łosiewicz (Institute of Media, Journalism and Social Communication, Faculty of Social Sciences, University of Gdańsk) [41,42].

### 2.5. Epidemiological Data from the Vaccination Surveillance System

In Poland, vaccination surveillance is conducted by the State Sanitary Inspectorate, which monitors vaccination implementation and compliance. Additionally, the Pharmaceutical Inspectorate supervises vaccine quality. The Chief Sanitary Inspector publishes the Immunization Program and is responsible for monitoring adverse events following immunization [43].

Vaccination data for children in Poland are collected by State District Sanitary Inspectorates and submitted annually to the National Institute of Public Health—National Institute of Hygiene—State Research Institute in Warsaw via the MZ-54 protective vaccination reporting form.

The State District Sanitary Inspector in Radomsko provided statistical data on measles and pertussis vaccinations among children born between 2014 and 2019 (children in grades 1–3).

The disclosed data included numbers of the following:

• Primary vaccinations;
• Booster doses;
• Unvaccinated individuals (for primary, catch-up, and booster immunizations).

### 2.6. Statistical Analysis

#### Questionnaire Survey

For the analysis, metric variables such as age and education level were utilized. Due to the predominance of women among respondents (94.99%), this variable was not used in further analyses. The level of knowledge about protective vaccinations was also used as a variable.

To estimate the level of knowledge about vaccinations, a new random variable (knowledge indicator) was constructed, taking values on a scale from 0 to 10 points. This variable was defined as the sum of points awarded according to established criteria for correct answers in the questionnaire. A respondent received one point for each answer demonstrating correct knowledge. A point was awarded, among other situations, when the respondent performed the following:

• Declared familiarity with the concept of protective vaccination and the mandatory vaccination schedule;
• Was able to name at least several (five or more) diseases covered by mandatory vaccinations and several recommended vaccinations;
• Was aware of potential consequences of not vaccinating children (e.g., increased risk of contracting diseases, resurgence of already eliminated diseases, threat to immunocompromised individuals);
• Knew possible, actual adverse effects of vaccines;
• Expressed belief in the benefits resulting from protective vaccinations (e.g., reduction in disease incidence, protection of society);
• Demonstrated familiarity with concepts such as herd immunity or eradication of infectious diseases (e.g., by indicating smallpox as an example of a disease fully eliminated thanks to vaccinations).

Based on the total score, respondents were assigned to one of three groups: low knowledge (0–4 points)—48 persons; medium knowledge (5–7 points)—234 persons; high knowledge (8–10 points)—177 persons.

This constructed indicator served for further statistical analyses aimed at assessing the relationship between the level of knowledge and the declared vaccination status of the respondents’ children against selected infectious diseases (measles, pertussis).

The study also utilized the Vaccine Attitudes Examination Scale (VAX) as a psychometric tool for identifying the level of acceptance, reluctance, or skepticism towards vaccinations in the population [41,42]. The questionnaire consisted of 12 statements to which respondents expressed their position on a Likert scale (from 1 “strongly disagree” to 6 “strongly agree”). The scale comprises four main subscales, each measuring a different aspect of reluctance or skepticism towards vaccinations:

- Mistrust of Vaccine Benefit—Measures the belief that vaccines are not effective or do not provide real benefits (e.g., “I am not convinced that vaccinations actually prevent diseases.”).
- Worries about Unforeseen Future Effects—Concerns fear of long-term, unknown effects of vaccinations (e.g., “I worry that vaccines may cause side effects that will only appear years later.”).
- Concerns about Commercial Profiteering—Refers to suspicions that vaccinations are promoted mainly for the profit of pharmaceutical companies and institutions (e.g., “Pharmaceutical companies promote vaccines only to make money.”).
- Preference for Natural Immunity—Expresses the belief that contracting the disease is better than receiving the vaccine (e.g., “I prefer to acquire immunity naturally rather than get vaccinated.”).

Each subscale consisted of three statements. To conduct further statistical analysis, a new variable (VAX1, VAX2, VAX3, VAX4) was created for each subscale by summing the responses given by the respondent to all three statements (range: 3–18 points). Reverse scoring was applied to the first subscale to standardize the direction of result interpretation.

Subsequently, to simplify interpretation and enable statistical analysis, each scale was dichotomized into two groups, creating four binary variables:

- Low vs. high mistrust in vaccine effectiveness (VAX_Mistrust);
- Low vs. high concerns about side effects (VAX_Worries);
- Low vs. high distrust of industry and authorities (VAX_Profiteering);
- Low vs. high preference for natural immunity (VAX_NaturalImmunity).

The dichotomization was performed using cut-off points determined based on data distribution:

• Low attitude level corresponded to a sum score of 3 to 10 points;
• High attitude level corresponded to 11–18 points.

These binary variables were then used in further statistical analyses, including testing associations with vaccination knowledge level and the vaccination status of respondents’ children.

For comparing categorical variables, Pearson’s chi-square test of independence was applied to assess associations between categorical variables in two independent groups. For table cells with low frequencies or zero values, Yates’s continuity-corrected chi-square test or Fisher’s exact test was used to ensure greater precision in small-sample analyses. The odds ratio (OR) with a 95% confidence interval (CI) was calculated to estimate the strength of associations between variables. The OR indicates the increased or decreased probability of an outcome occurring in one group relative to another. A *p*-value <0.05 was adopted as the criterion for statistical significance. All analyses were performed using R software version 4.4.1.

### 2.7. Reliability Analysis

Internal consistency of the VAX subscales was assessed using Cronbach’s alpha (α) and Guttman’s lambda-6 (G6) coefficients. Both indicators are commonly used to evaluate the internal reliability of multi-item scales, with G6 providing a more conservative estimate by accounting for item-specific variance (Table 2).

All subscales reached Cronbach’s alpha values close to or exceeding 0.90, which indicates a high level of psychometric reliability. In particular, the Mistrust subscale demonstrated the strongest internal consistency, suggesting a high degree of agreement among respondents in evaluating the perceived benefits of vaccination. The additional reliability indicator G6 (lambda 6) also supports the internal coherence of the subscales, with values above 0.80 across all dimensions.

Moreover, the reliability analysis confirmed that the overall VAX scale also demonstrated very good internal consistency, with α = 0.94, G6 = 0.96, and a 95% confidence interval for alpha of [0.932; 0.948]. These results further support the internal coherence of the scale as a whole and indicate that the aggregated measure captures a consistent pattern of attitudes toward vaccination [44].

### 2.8. Epidemiological Data

Based on the obtained data, differences in measles and pertussis vaccination gaps were analyzed. Statistical analysis of vaccine refusal rates was performed using the Z-test for two proportions. This test compares the difference between two independent proportions, assuming dichotomous data (vaccinated vs. unvaccinated child). Vaccination refusal rates were calculated using the formula: (number of unvaccinated children/number of children in each birth cohort subject to vaccination mandate) × 100%. A proportion test was then applied to assess the statistical significance of differences between vaccination refusal rates across different years. Calculations were performed under the assumption of independent and sufficiently large samples according to the Z-test conditions. The test results enabled evaluation of whether observed differences in child vaccination coverage between specific years were statistically significant, allowing conclusions about the directions and dynamics of vaccination refusal trends during the analyzed period.

## 3. Results

### 3.1. Characteristics of the Study Group

The study involved 459 individuals aged 24 to 63 years (mean: M = 38.9, standard deviation: SD = 5.6). Women predominated in the study group (94.99%). Regarding education, a marked dominance of individuals with a master’s-level education (67.10%) was observed, reflecting the current educational structure in Poland. Over 75% of respondents rated their financial situation as average. Nearly 90% of respondents were employed individuals. Most respondents had only one child, and none had more than two children (Table 3).

### 3.2. Knowledge About Protective Vaccinations

The majority of respondents correctly identified measles and pertussis as diseases covered by mandatory vaccinations in Poland (92.37% vs. 85.19%, respectively).

Nearly 80% of respondents erroneously classified pneumococcal and rotavirus vaccinations as recommended, while only about 1% indicated the correct answer (‘mandatory’) (1.09% for pneumococcus vs. 0.87% for rotavirus). Simultaneously, 77.78% of participants believed varicella vaccination is mandatory in Poland. Only 37.47% of respondents considered hepatitis A (WZW A) vaccination as recommended.

For almost 50% of participants, the primary benefit of vaccinations was reducing disease severity. Over 5% believed vaccinations provide no benefits.

Regarding potential consequences of non-vaccination, most parents (76.03%) selected ‘increased risk of disease complications’. As many as 11.5% perceived no negative consequences of vaccine refusal.

The most frequently recognized vaccine side effect was fever (reported by >87%). Awareness of measles complications was limited; only 45% correctly identified encephalitis as a serious complication. For pertussis, >56% recognized key complications (respiratory failure and bacterial pneumonia), while knowledge of pertussis encephalopathy remained low (17%). Response distributions are presented in Table 4.

Parents with higher education demonstrated significantly better knowledge regarding protective vaccinations (particularly concerning vaccination awareness, herd immunity, MMR, eradicated diseases, and public health significance) and were considerably more open to administering non-mandatory, recommended vaccinations. Compliance with the mandatory vaccination schedule was high in both groups (96.1% for higher education vs. 91.2%). The results indicated that education level was a significant factor in detailed knowledge and attitudes toward non-compulsory vaccinations (e.g., HPV vaccination) but did not determine basic compliance with the mandatory immunization program. Educational initiatives should focus on enhancing knowledge about herd immunity, historical vaccination successes (such as smallpox eradication), and benefits of non-mandatory vaccinations, particularly among parents with lower education levels (Table 5).

We observed a statistically significant association (*p* < 0.05) between parental knowledge level and vaccination decisions for their children. As parental knowledge increased, vaccination coverage against both diseases (measles and pertussis) rose (14.6% [low], 38.0% [medium], and 72.3% [high], respectively). Interest in non-mandatory (recommended) vaccinations also increased with parental knowledge level—from 20.8% (low knowledge) to 69.5% (high knowledge) (Table 6). A statistically significant relationship was observed between vaccination knowledge level and both material situation (*p* = 0.0098) and employment status (*p* < 0.0001). Notably, none of the respondents with a high material situation were classified in the low knowledge group, and none of the respondents indicating being in education as their employment status were found in the low knowledge group.

### 3.3. Attitudes Towards Vaccinations

The mean scores for the VAX subscales indicated varying levels of vaccine skepticism. The lowest averages were observed for VAX_1 (mistrust of vaccine benefits; M = 7.60, SD = 3.81, 95% CI: [7.25; 7.95]) and VAX_4 (preference for natural immunity; M = 7.40, SD = 4.13, 95% CI: [7.02; 7.78]), suggesting moderate mistrust in these areas. A notably higher score was found for VAX_2 (worries about side effects; M = 11.81, SD = 4.11, 95% CI: [11.43; 12.19]), reflecting stronger safety concerns. VAX_3 (distrust of the pharmaceutical industry) yielded M = 7.71 (SD = 4.20, 95% CI: [7.33; 8.10]). Observed response variability (high SDs and differing modes) highlights substantial attitude heterogeneity, underscoring the need for segmented vaccine communication strategies.

**Figure 4:**
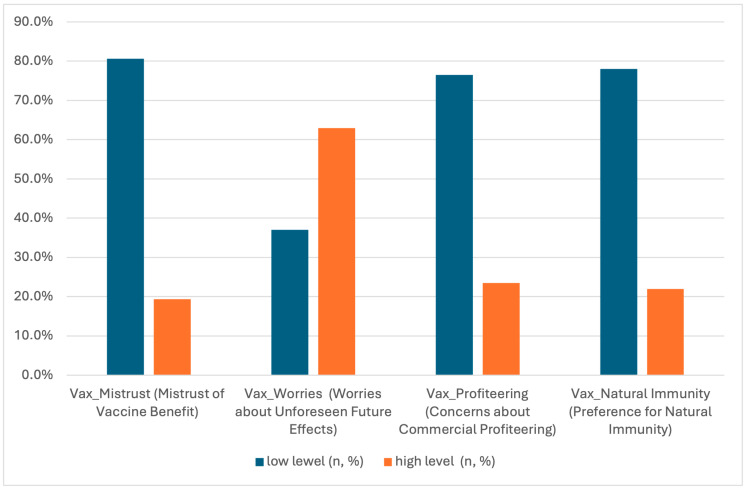
Attitudes towards vaccinations according to the VAX scale.

The highest percentage of respondents (80.6%) exhibited low levels of mistrust toward vaccine benefits, while concerns about unforeseen future effects were most prevalent, affecting 63.0% of participants (Figure 4).

Parental education significantly influenced child vaccination decisions, particularly for pertussis vaccination (OR = 2.03; *p* < 0.001) and combined measles/pertussis vaccination (OR = 1.83; *p* < 0.001). Parents with higher education were more likely to vaccinate according to recommendations (OR = 2.37; *p* = 0.067).

Knowledge level was a key motivator of vaccination decisions, especially in areas requiring informed choice. In our study, the parental knowledge index showed strongest correlation with pertussis vaccination (OR = 9.64; *p* < 0.001) and combined measles/pertussis vaccination (OR = 9.08; *p* < 0.001). Knowledge significantly correlated with other proactive behaviors: uptake of recommended vaccines (OR = 4.24; *p* < 0.001) and adherence to the Immunization Program schedule (OR = 3.92; *p* < 0.001). Surprisingly, knowledge showed no association with the completion of mandatory vaccinations (OR = 1.44; *p* = 0.43) (Table 7).

**Table 7:** Impact of parental education and knowledge index on child vaccination completion—statistical analysis (OR, 95% CI, *p*-value).

Variable	Type of Vaccine/Behavior	OR	95% CI	Statistical Significance
Education				
	Measles	1.10	0.68–1.76	0.73
	Pertussis	2.03	1.27–3.27	0.0023
	Measles + Pertussis	1.83	1.14–2.97	0.0097
	All mandatory vaccinations	2.37	0.87–6.08	0.067
Knowledge Index				
	Adherence to vaccination schedule	3.92	2.13–7.20	4.49 × 10^−6^
	Acceptance of additional (recommended) vaccinations	4.24	2.47–7.53	1.14 × 10^−8^
	Measles	2.84	1.73–4.68	1.40 × 10^−5^
	Pertussis	9.64	5.17–19.19	2.50 × 10^−17^
	Measles + Pertussis	9.08	4.71–18.96	1.44 × 10^−15^
	All mandatory vaccinations	1.44	0.45–3.96	0.43

The study demonstrated a statistically significant association between negative parental attitudes (high mistrust, serious concerns, strong vaccine-hesitant preferences) and low completion rates of both mandatory and recommended childhood vaccinations. Among parents characterized by low mistrust, minimal concerns, low vaccine anxiety, and weak anti-vaccination preferences, the proportion of children vaccinated according to the official immunization schedule was substantially higher compared to groups with opposing attitudes. The most pronounced differences concerned completion of all mandatory vaccinations; only 75.3% of children of high-mistrust parents were fully vaccinated, versus 99.7% in the low-mistrust group (OR = 0.0084; *p* < 0.000001). Similarly, in groups with serious concerns and strong vaccine-hesitant preferences, only ≈80% of children received all mandatory vaccinations, whereas corresponding comparison groups achieved 99–100% coverage.

Additionally, parents with negative attitudes less frequently considered recommended vaccinations and more frequently declared intent to forgo future vaccinations (OR≈0.05, *p* < 0.001) compared to more positively inclined groups (Table 8).

Parents aged ≥39 years more frequently reported full completion of mandatory vaccinations for their children (97.4% vs. 93.3%, OR = 2.67; *p* = 0.04). No differences were observed regarding measles and pertussis vaccinations. Notably, parents in both age groups equally considered non-mandatory vaccinations for their children (OR = 1.26; *p* = 0.13) and planned future vaccinations according to the schedule (OR = 1.15; *p* = 0.36), suggesting similar levels of proactive attitudes in these aspects regardless of age (Table 9).

Regarding access to information and attitudes regarding vaccinations, only every third respondent knew that schools were involved in vaccination promotion (32%, N = 149). Less than half of respondents (44%, N = 206) considered vaccination-related information sufficiently accessible. Over half believed schools should be more engaged in health education. As many as 61% (N = 281) reported knowing individuals in their social circle who do not vaccinate their children (Table 10). Among non-vaccinating parents (n = 18), the primary reason cited was negative experiences with previous vaccinations (n = 15). Other reported reasons included vaccine safety concerns (n = 1), child health issues (n = 1), and insufficient support from healthcare professionals (n = 1).

### 3.4. Epidemiological Statistical Data—Analysis of Vaccine Refusals in Radomsko County and Radomsko City, 2014–2019

**Figure 5:**
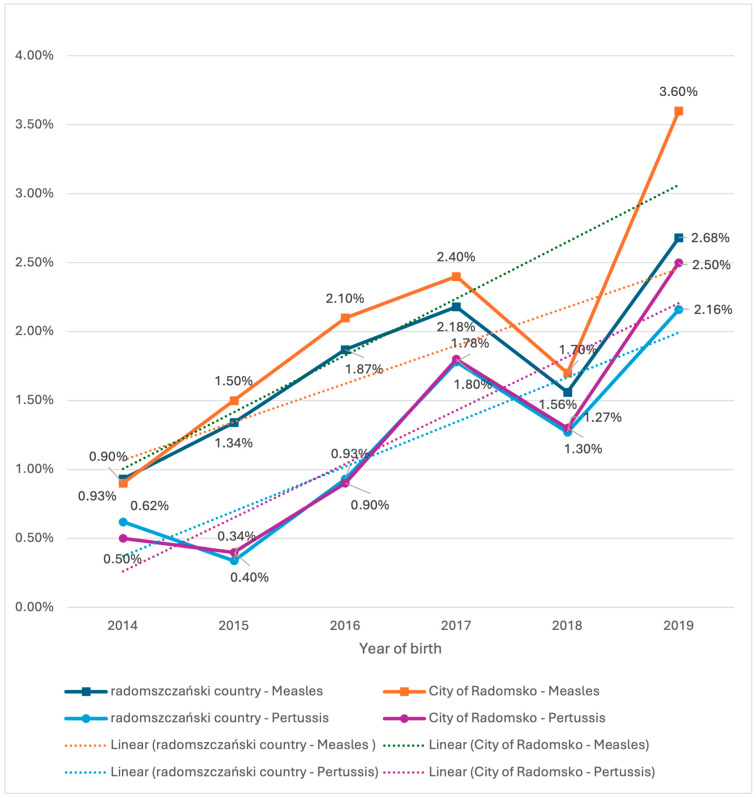
Vaccination Refusal Trends in Radomszczański Country and Radomsko City (2014–2019).

During the analyzed period (2014–2019), a gradual increase in the refusal rate of protective vaccinations was observed. A notable rise of 2.7 percentage points occurred specifically for measles vaccine refusals in Radomsko City (Figure 5).

## 4. Discussion

Parental knowledge and opinions constitute a key factor influencing decisions regarding child vaccination. Risk perception, trust in the healthcare system, quality of received information, and level of vaccination knowledge substantially determine parental health-promoting behaviors [45,46].

The aim of our study was to assess the knowledge and opinions of parents of children aged 6–11 years from primary schools in Radomsko toward protective vaccinations, with particular emphasis on vaccinations against measles and pertussis, and to evaluate vaccination coverage against these diseases. We revealed substantial disparities in vaccination practices and knowledge among parents of children from Radomsko. Despite declared awareness of the existence of protective vaccinations (96.95%), practical familiarity with the Immunization Program (PSO) remained low, with only 73.4% of respondents claiming full knowledge on this subject. Recognition of diseases covered by mandatory vaccinations in Poland was insufficient (e.g., pneumococcus: 1.09%; rotavirus: 0.87%). Education proved to be a key determinant for knowledge—parents with higher education demonstrated significantly better understanding of herd immunity concepts (91% vs. 71.6%) and smallpox eradication history (16.8% vs. 8.8%), which confirms findings that education level is strongly correlated with general health literacy and the capacity to comprehend complex medical issues, including vaccine mechanisms and their role in public health. Similar relationships were observed in another study conducted in Poland, which indicated that parental education influenced the decision to choose combined vaccines—despite certain differences in risk perception [47]. International studies, such as that by Johns et al. (2022), analyzed the impact of maternal education level and economic status on vaccination completion in South Asia, demonstrating a clear association between higher maternal education and greater likelihood of full vaccination of offspring [48].

Despite generally high declared adherence to the vaccination schedule (96.1% in the higher education group, 91.2% in others), concerning patterns were observed in vaccination completion against specific diseases. Only 60.8% of children in the <39-year-old parent group were vaccinated against measles and against pertussis—55.2%. Results were particularly alarming in low-knowledge groups; merely 14.6% of these parents’ children received vaccinations against both measles and pertussis. Paradoxically, while knowledge strongly influenced acceptance of recommended vaccinations (OR = 4.24; *p* < 0.001), it did not determine mandatory vaccinations (OR = 1.44; *p* = 0.43), suggesting a dominant role of systemic factors (legal mandate, accessibility) for compulsory immunizations.

Analysis of VAX scale results revealed a profound impact of multidimensional parental attitudes on vaccination practices. In the high-mistrust group, only 0.3% of children received all mandatory vaccinations, versus 99.7% in the low-mistrust group (OR = 0.0084; *p* < 0.000001). This contrast confirms findings by Martin and Petrie (2017), who in the original VAX scale validation demonstrated that mistrust of vaccine benefits is the strongest predictor of vaccination refusals [49].

Alarmingly, 11.5% of respondents perceived no negative consequences of vaccine refusal, and 5% denied vaccination benefits. These attitudes are reflected in the increasing trend of vaccination refusals in Radomsko (+2.7 pp for measles during 2014–2019), representing a local manifestation of global institutional distrust, where lack of trust in the healthcare system correlates with lower vaccination acceptance—consistent with Sturm et al. (2021) regarding influenza vaccination during the COVID-19 pandemic [50].

Parents may perceive vaccinations as a potential threat to child health, despite lacking scientific evidence. Safety concerns—particularly regarding adverse events following immunization—evoke especially strong emotions. Although serious vaccine adverse events are extremely rare (estimated at 1 case per million doses), these fears remain deeply settled in parental consciousness. They often involve alleged complications such as autism, allergies, or other neurological problems—despite these associations being unequivocally refuted by numerous scientific studies [51]. The presence and activities of anti-vaccine movements in Poland also influence parental attitudes. Familiarity with arguments promoted by these groups may lead to increased vaccination refusals—including for mandatory vaccines. In our study, two schools declined survey participation, deeming the topic ‘controversial’, reflecting societal tensions surrounding vaccinations.

Insufficient health education and growing distrust toward medical institutions constitute additional aggravating factors. Research by Wojtyczka et al. (2022) demonstrated that inadequate health literacy significantly correlated with lower vaccine acceptance [52]. Conversely, Rodriguez et al. (2023) proved that parental anxiety—particularly maternal—and distrust in medical institutions are significant predictors of childhood vaccine refusal [53].

While online information accessibility constitutes an undoubted advantage, it simultaneously creates a platform for spreading misinformation. Social media have become primary channels for propagating myths about alleged vaccine harm—such as links between vaccinations and autism, dangerous vaccine ingredients, or beliefs about pharmaceutical industry profits [54]. The COVID-19 pandemic significantly limited access to direct medical consultations, leading parents to increasingly use the internet as their main knowledge source, often without opportunities for expert verification. This trend is particularly concerning since medical professionals (physicians, nurses) and educational institutions should constitute the primary and most trusted sources of reliable information. According to a UNICEF report (2023), 52 out of 55 analyzed countries recorded decreased confidence in childhood vaccinations post-pandemic, with the most significant declines occurring in South Korea, Papua New Guinea, Ghana, Senegal, and Japan [55]. In our study, only 32% of parents were aware of schools’ engagement in vaccination promotion activities. This result raises critical questions about the source of this limited awareness: whether it stems from insufficient parental interest in vaccination topics or inadequate communication from educational institutions. Likely, both parties require enhanced support in establishing effective information channels and strengthening collaboration in health education. Reinforcing these initiatives may, in the long-term perspective, contribute to improved societal health awareness and consequently enhance public health.

The WHO European Region implemented the Measles Elimination Program in 2001, aiming to eradicate the pathogen across 53 countries [34]. This study’s results revealed that key barriers to achieving this goal in Poland—particularly gaps in knowledge about the Polish Immunization Program and vaccine-hesitant attitudes—were deeply entrenched at the local level. As indicated by Mucha et al. (2025), successful elimination requires coordinated educational interventions addressing regional behavioral patterns, especially concerning misconceptions about ‘natural immunity’ [54] (observed in 83.33% of non-vaccinating parents in Radomsko). Without targeted community-level interventions in populations like Radomsko residents, where mistrust intensity exceeds national averages, the achievement of the WHO’s 2030 targets may be severely compromised.

Our survey identified a distinct socio-demographic profile associated with elevated child non-vaccination risk. The high-risk parent was characterized primarily by profound vaccine mistrust—the strongest refusal predictor. In this group, merely 0.3% of children had received all mandatory vaccinations. This mistrust manifested in the following ways:

• Failure to perceive consequences of non-vaccination (11.5% of respondents);
• Denial of vaccine benefits (5%);
• Strong belief in ‘natural immunity’ (observed in 83.33% of non-vaccinators).

This profile was further compounded by the following:

• Healthcare knowledge deficits, particularly regarding the Immunization Program (only 73.4% claimed familiarity) and vaccine-preventable diseases.
• Younger age (<39 years)—The group with lowest vaccination coverage against measles (60.8%) and pertussis (55.2%). This may correlate indirectly with higher susceptibility to vaccine misinformation on social media, as confirmed by other studies [34,56].
• Lower education levels—Significantly associated with limited comprehension of key concepts (herd immunity, public health).

This study also presents several significant limitations requiring consideration during results interpretation. Firstly, the obtained data cannot be generalized to the entire population of parents in Poland. Moreover, our sample is a convenience sample of parents who voluntarily responded to the survey. This may introduce a selection bias, as it is likely that individuals for whom the topic of vaccinations is particularly important or controversial were more inclined to participate. The research was conducted in one mid-sized city (Radomsko), and vaccination attitudes and behaviors may vary depending on place of residence, local cultural norms, and healthcare accessibility.

Secondly, although the VAX scale used underwent preliminary validation for internal consistency and construct validity within the study group, the survey methodology was exclusively online. This may introduce selection bias risk—participation required internet access, basic digital literacy, and willingness to discuss vaccinations. Consequently, individuals with stronger opinions (both pro- and anti-vaccination) may have been disproportionately represented, potentially compromising sample representativeness.

Additionally, the study relied on self-reported data, carrying risks of social desirability bias and recall errors—particularly for questions concerning personal vaccination experiences or self-assessed knowledge levels.

Future research should consider geographically diversified sampling (e.g., large urban and rural areas), employing mixed methods approaches (quantitative and qualitative) with direct recruitment to better capture the full spectrum of attitudes and knowledge levels present in the general population.

## 5. Conclusions

Substantial parental knowledge gaps regarding vaccination programs and diseases, coupled with significant mistrust, constitute critical barriers to optimal child vaccination coverage in Radomsko. These factors, particularly prevalent among lower-education groups, correlate strongly with declining measles and pertussis vaccination rates. This trend poses a direct threat to achieving national disease elimination goals (e.g., measles by 2030) and controlling pertussis resurgence. Addressing this requires systemic, evidence-based health education strategies. Priorities include personalized risk communication targeting vulnerable groups and leveraging schools for public health promotion to counteract declining coverage and rebuild vaccine confidence.

## Figures and Tables

**Table 1:** Immunization Program in Poland (2025) [9].

Vaccine	Mandatory	Recommended
Tuberculosis (BCG)	✓	
Hepatitis B	✓	
Diphtheria, Tetanus, Pertussis (DTP)	✓	
Haemophilus influenzae type b (Hib)	✓	
Poliomyelitis	✓	
Rotavirus	✓	
Pneumococcal	✓	
Measles, Mumps, Rubella (MMR)	✓	
Human Papillomavirus (HPV)		✓ *
COVID-19		✓ **
Meningococcal		✓
Varicella (Chickenpox)		✓
Tick-borne Encephalitis (TBE)		✓
Hepatitis A		✓
Influenza		✓ ***

* Publicly funded for individuals aged 9–14 years. Reimbursement available for one vaccine product for those aged 9–18 years; ** publicly funded; *** 100% reimbursement: children (6 months–18 years), persons ≥65 years, and pregnant women; 50% reimbursement: individuals aged 18–64 years.

**Table 2:** Results of the reliability analysis conducted in R indicate a high internal consistency for each of the four VAX subscales:.

Subscale	Cronbach’s α	95% Confidence Interval for α	Lambda 6 (G6)
Mistrust	0.96	[0.954; 0.966]	0.94
Worries	0.89	[0.873; 0.907]	0.84
Natural Immunity	0.91	[0.893; 0.927]	0.88
Profiteering	0.91	[0.894; 0.926]	0.87
Overall VAX	0.94	[0.932; 0.948]	0.96

**Table 3:** Sociodemographic characteristics of the study participants.

Variable	N (%)
Sex	
Male	19 (4.14)
Female	436 (94.99)
Prefer not to say	4 (0.87)
Age group (years)	
24–33	85 (18.50)
34–43	270 (58.80)
44–53	101 (22.0)
54–63	3 (0.70)
Education level	
Primary	12 (2.61)
Secondary	90 (19.61)
Bachelor’s	43 (9.37)
Master’s	308 (67.10)
PhD or higher	6 (1.31)
Socioeconomic status	
Low income	49 (10.68)
Middle income	353 (76.91)
High income	57 (12.42)
Employment status	
Unemployed	42 (9.15)
Employed	405 (88.24)
Student	12 (2.61)
Number of children	
1	403 (87.80)
2	56 (12.20)

**Table 4:** Vaccination knowledge and attitudes among study participants.

	N (%)
Are you familiar with what a protective vaccination is?	
Yes	445 (96.95)
No	1 (0.22)
Not sure	13 (2.83)
Please indicate which diseases are covered by mandatory vaccinations in Poland (multiple selections allowed)	
Measles *	424 (92.37)
Pertussis *	391 (85.19)
Poliomyelitis *	335 (72.98)
Varicella **	171 (37.25)
Diphtheria *	357 (77.78)
Tetanus *	366 (79.74)
Hepatitis B *	291 (63.40)
Hepatitis A **	61 (13.29)
Pneumococci *	5 (1.09)
Rotaviruses *	4 (0.87)
Don’t know	6 (1.31)
Please indicate which diseases have vaccines recommended (but not mandatory) in Poland (multiple selections allowed)	
Influenza **	366 (79.74)
Human papillomavirus **	356 (77.56)
Rotaviruses *	359 (78.21)
Pneumococci *	367 (79.96)
Meningococci **	329 (71.68)
Tick-borne encephalitis **	242 (52.72)
Varicella **	276 (60.13)
Hepatitis A **	172 (37.47)
Don’t know	23 (5.01)
Please select the most important benefit of vaccinations (choose one primary answer)	
Protection against infectious diseases	165 (35.90)
Protection of individuals who cannot be vaccinated (herd immunity)	1 (0.22)
Reduction of epidemic risk	43 (9.37)
Reduction of disease severity if infected (less hospitalization/death)	225 (49.02)
In my view, vaccinations provide no meaningful benefits	24 (5.23)
Please indicate potential consequences of not vaccinating a child (multiple selections allowed)	
Higher risk of disease complications	349 (76.03)
Increased likelihood of infection	318 (69.28)
Risk to other children	215 (46.84)
Possible exclusion from school/preschool	63 (13.73)
In my view, there are no negative consequences of not vaccinating children	53 (11.55)
Please indicate what the most common side effects of vaccinations are (multiple selections allowed)	
Pain at the injection site	366 (79.74)
Fever	400 (87.15)
Rash	126 (27.45)
Fatigue	146 (31.81)
Excessive crying	57 (12.42)
Have you heard of the MMR vaccine (measles, mumps, rubella)?	
Yes	403 (87.80)
No	56 (12.20)
Please specify which complications may occur following measles infection (multiple selections allowed)	
Cutaneous manifestation	114 (24.84)
Encephalitis	208 (45.32)
Pneumonia	127 (27.67)
Ocular complications	86 (18.74)
Don’t know	168 (36.60)
Please enumerate the potential complications associated with pertussis infection (multiple selections allowed)	
Paroxysmal coughing spells	231 (50.33)
Respiratory failure	260 (56.64)
Bacterial pneumonia	260 (56.64)
Pertussis encephalopathy	78 (16.99)
Don’t know	87 (18.95)

* mandatory vaccinations; ** vaccines recommended.

**Table 5:** Comparison of vaccination knowledge, attitudes, and practices by parental education level.

Answers to the Questionnaire Questions	Education Level		Statistical Significance
	Primary/Secondary n = 102 N (%)	Higher Education n = 357 N (%)	
Do you know what a preventive (protective) vaccination is?			
Yes	89 (87.30)	356 (99.70)	*p* = 8.74 × 10^−10^
No	1 (1.00)	0 (0.00)	
I’m not sure	12 11.80)	1 (0.30)	
Are you familiar with the schedule of mandatory vaccinations in Poland?			
Yes	75 (73.50)	262 (73.40)	*p* = 0.642
No	5 (4.90)	11 (3.10)	
I am aware of it, but I am not familiar with the details	22 (2.16)	84 (23.50)	
Have you heard of the MMR vaccine (measles, mumps, rubella)?			
Yes	84 (82.40)	319 (98.40)	*p* = 0.0567
No	18 (17.60)	39 (10.60)	
Do you know why vaccination are important for public health?			
Yes	87 (85.30)	328 (91.9)	*p* = 0.0305
No	5 (4.90)	4 (1.10)	
I’m not sure	10 (9.80)	25 (7.00)	
Do you know what herd/community immunity is?			
Yes	73 (71.60)	325 (91.00)	*p* = 2.10 × 10^−6^
No	4 (3.90)	5 (1.40)	
I am aware of it, but I am not familiar with the details.	25 (24.50)	27 (7.60)	
Which disease has been eradicated worldwide thanks to vaccinations?			
Accurate response	9 (8.80)	60 (16.80)	*p* = 0.0466
Inaccurate response	93 (91.20)	297 (83.20)	
Has your child received all mandatory vaccinations?			
Yes	93 (91.20)	343 (96.10)	*p* = 1.43 × 10^−4^
No	4 (3.90)	14 (3.90)	
I’m not sure	5 (4.90)	0 (0.00)	
If your child had adverse reactions after vaccination, were they reported to a doctor?			
Yes	12 (63.20)	35 (85.40)	*p* = 0.0521
No	7 (36.80)	6 (14.60)	
Do you plan a vaccinate your child in the future according to the vaccination schedule?			
Yes	87 (85.30)	310 (86.80)	*p* = 0.774
No	6 (5.90)	15 (4.20)	
I have not made a decision yet	9 (8.80)	32 (9.00)	
Are you considering additional vaccinations for your child (e.g., against flu, HPV)?			
Yes	31 (30.40)	201 (56.30)	*p* = 2.92 × 10^−7^
No	27 (26.50)	89 (24.90)	
I have not made a decision yet	44 (43.10)	67 (18.80)	

**Table 6:** Comparison of vaccination knowledge, attitudes, and practices by parental knowledge level.

Answers to the Questionnaire Questions	Knowledge Level			Statistical Significance
	Low Knowledge n = 48 N (%)	Moderate Knowledge n = 234 N (%)	High Knowledge n = 177 N (%)	*p*-Value
Has your child been vaccinated against measles?				
Vaccinated	21 (43.80)	114 (48.70)	143 (80.80)	*p* = 1.57 × 10^−11^
Not vaccinated	27 (56.20)	120 (51.30)	34 (19.20)	
Has your child been vaccinated against pertussis?				
Vaccinated	8 (16.70)	101 (43.20)	138 (78.00)	*p* = 7.47 × 10^−18^
Not vaccinated	40 (83.30)	133 (56.80)	39 (22.00)	
Has your child been vaccinated against both measles and pertussis?				
Vaccinated against both	7 (14.60)	89 (38.00)	128 (72.30)	*p* = 1.78 × 10^−16^
Other	41 (85.40)	145 (62.00)	49 (27.70)	
Has your child received all mandatory vaccinations?				
Yes, received all mandatory vaccinations	47 (97.90)	213 (91.00)	176 (99.40)	*p* = 3.46 × 10^−4^
No, did not receive all mandatory vaccinations	1 (2.10)	21 (9.00)	1 (0.60)	
Do you plan a vaccinate your child in the future according to the vaccination schedule?				
Yes	39 (81.20)	183 (78.20)	175 (98.80)	*p* = 5.34 × 10^−9^
No	9 (18.80)	51 (21.80)	2 (1.10)	
Are you considering additional vaccinations for your child (e.g., against flu, HPV)?				
Yes	10 (20.80)	99 (42.30)	123 (69.50)	*p* = 2.63 × 10^−11^
No	38 (79.20)	135 (57.70)	54 (30.50)	

**Table 8:** Association between parental attitudes towards vaccinations and child vaccination schedule completion (mandatory and recommended)—comparative analysis stratified by mistrust, worries, concerns, and preference.

**Variable VAX_1**	**Low Mistrust** **n = 370** **N (%)**	**High Mistrust** **n = 89** **N (%)**	**OR**	**95% CI**	**Statistical** **Significance**
Has your child received all mandatory vaccinations?					
Yes	369 (99.70)	67 (75.30)	0.0084	0.00021–0.05350	4.081131 × 10^−16^
No	1 (0.30)	22 (24.70)			
Has your child been vaccinated against measles?					
Vaccinated	243 (65.70)	35 (39.30)	0.5599	0.20387–0.5599	6.848 × 10^−6^
Not vaccinated	127 (34.30)	54 (60.70)			
Has your child been vaccinated against pertussis?					
Vaccinated	215 (58.10)	32 (36.00)	0.4055	0.24209–0.6709	0.0002186
Not vaccinated	155 (41.90)	57 (64.00)			
Has your child been vaccinated against measles and pertussis?					
Vaccinated	197 (53.20)	27 (30.30)	0.3832	0.22377–0.64266	0.0001382
Not vaccinated	173 (46.80)	62 (69.70)			
Education Level					
Primary/Secondary	84 (22.70)	18 (20.20)	1.1582	0.63951–2.18373	0.672
Higher education	286 (77.30)	71 (79.80)			
Are you considering additional vaccinations for your child (e.g., against flu, HPV)?					
Yes	227 (61.40)	5 (5.60)	0.03772	0.011652–0.09478	<2.2 × 10^−16^
No	143 (38.60)	84 (94.40)			
Do you plan a vaccinate your child in the future according to the vaccination schedule?					
Yes	362 (97.80)	35 (39.30)	0.1459	0.00552–0.03397	<2.2 × 10^−16^
No	8 (2.20)	54 (60.70)			
**Variable VAX_2**	**Minor Worry** **n = 170** **N (%)**	**Severe Worry** **n = 289** **N (%)**	**OR**	**95% CI**	**Statistical** **significance**
Has your child received all mandatory vaccinations?					
Yes	170 (100.00)	266 (92.00)	n.a.	n.a.	0.000023
No	0 (0.00)	23 (8.00)			
Has your child been vaccinated against measles?					
Vaccinated	136 (80.00)	142 (49.10)	4.1277	2.61592–6.6392	3.988 × 10^−11^
Not vaccinated	34 (20.00)	147 (50.90)			
Has your child been vaccinated against pertussis?					
Vaccinated	117 (68.80)	130 (45.00)	2.6940	1.77982–4.11218	7.168 × 10^−7^
Not vaccinated	53 (31.20)	159 (55.00)			
Has your child been vaccinated against measles and pertussis?					
Vaccinated	114 (67.10)	110 (38.10)	3.3036	2.18362–5.03904	1.768 × 10^−9^
Not vaccinated	56 (32.90)	179 (61.90)			
Education Level					
Primary/Secondary	28 (16.50)	74 (25.60)	1.7435	1.05397–2.94501	0.02697
Higher education	142 (83.50)	215 (25.60)			
Are you considering additional vaccinations for your child (e.g., against flu, HPV)?					
Yes	125 (73.50)	107 (37.00)	4.7074	3.05792–7.3397	2.486 × 10^−14^
No	45 (26.50)	182 (63.00)			
Do you plan a vaccinate your child in the future according to the vaccination schedule?					
Yes	168 (98.80)	229 (79.20)	21.9237	5.6678–187.3108	4.978 × 10^−11^
No	2 (1.20)	60 (20.80)			
**Variable VAX_3**	**Mild Concerns** **n = 352** **N (%)**	**Severe Concerns** **n = 108** **N (%)**	**OR**	**95% CI**	**Statistical** **significance**
Has your child received all mandatory vaccinations?					
Yes	349 (99.40)	87 (80.60)	0.024	0.002268–0.10109	2.1 × 10^−12^
No	2 (0.60)	21 (19.40)			
Has your child been vaccinated against measles?					
Vaccinated	229 (65.20)	49 (45.40)	0.4433	0.2787–0.70220	0.0002968
Not vaccinated	122 (34.80)	59 (54.60)			
Has your child been vaccinated against pertussis?					
Vaccinated	215 (61.30)	32 (29.60)	0.2671	0.1617–0.4336	1.093 × 10^−8^
Not vaccinated	136 (38.70)	76 (70.40)			
Has your child been vaccinated against measles and pertussis?					
Vaccinated	193 (55.00)	31 (28.70)	0.3304	0.1996–0.5369	1.794 × 10^−6^
Not vaccinated	158 (45.00)	77 (71.30)			
Education Level					
Primary/Secondary	72 (20.50)	30 (27.80)	0.6716	0.39998–1.14415	0.1144
Higher education	279 (79.50)	78 (72.20)			
Are you considering additional vaccinations for your child (e.g., against flu, HPV)?					
Yes	213 (60.70)	19 (17.60)	0.1389	0.07632–0.24217	1.005 × 10^−15^
No	138 (39.30)	89 (82.40)			
Do you plan a vaccinate your child in the future according to the vaccination schedule?					
Yes	337 (96.00)	60 (55.60)	0.0525	0.0251–0.1038	<2.2 × 10^−16^
No	14 (4.00)	48 (44.40)			
**Variable VAX_4**	**Weak** **Preferences** **n = 358** **N (%)**	**Strong** **Preferences** **n = 101** **N (%)**	**OR**	**95% CI**	**Statistical** **significance**
Has your child received all mandatory vaccinations?					
Yes	355 (99.20)	81 (80.20)	0.346	0.0064–0.1206	1.423 × 10^−11^
No	3 (0.80)	20 (19.80)			
Has your child been vaccinated against measles?					
Vaccinated	228 (63.70)	50 (49.50)	0.5597	0.34938–0.89563	0.0113
Not vaccinated	130 (36.30)	51 (50.50)			
Has your child been vaccinated against pertussis?					
Vaccinated	213 (59.50)	34 (33.70)	0.3463	0.2105–0.5617	5.147 × 10^−6^
Not vaccinated	145 (40.5)	67 (66.30)			
Has your child been vaccinated against measles and pertussis?					
Vaccinated	190 (53.10)	34 (33.70)	0.4495	0.2737–0.7280	0.0006825
Not vaccinated	168 (46.90)	67 (66.30)			
Vaccination Knowledge					
Low vaccination knowledge	32 (8.90)	16 (15.80)	0.5223	0.2637–1.0695	0.06366
Advanced vaccination knowledge	326 (91.10)	85 (84.20)			
Education Level					
Primary/Secondary	66 (18.40)	36 (35.60)	0.4090	0.2447–0.6880	0.0004033
Higher education	292 (81.60)	65 (64.40)			
Are you considering additional vaccinations for your child (e.g., against flu, HPV)?					
Yes	215 (60.10)	17 (16.80)	0.1352	0.07208–0.24113	5.657 × 10^−15^
No	143 (39.90)	84 (83.20)			
Do you plan a vaccinate your child in the future according to the vaccination schedule?					
Yes	3 (95.80)	54 (53.50)	0.0508	0.02456–0.09988	<2.2 × 10^−16^
No	15 (4.20)	47 (46.50)			

n.a.—not applicable

**Table 9:** Association between parental age groups and vaccination practices, knowledge, and attitudes toward protective vaccinations (N = 459).

Variable	Age (<39) n = 268 N (%)	Age (≥39) n = 191 N (%)	OR	95% CI	Statistical Significance
Has your child received all mandatory vaccinations?					
Yes	250 (93.30)	186 (97.40)	2.67	0.934–9.383	0.04
No	18 (6.70)	5 (2.60)			
Has your child been vaccinated against measles?					
Vaccinated	163 (60.80)	115 (60.20)	0.97	0.655–1.452	0.59
Not vaccinated	105 (39.20)	76 (39.80)			
Has your child been vaccinated against pertussis?					
Vaccinated	148 (55.20)	99 (51.80)	0.87	0.591–1.288	0.79
Not vaccinated	120 (44.80)	92 (48.20)			
Has your child been vaccinated against measles and pertussis?					
Vaccinated	131 (48.90)	93 (48.70)	0.99	0.673–1.464	0.6
Not vaccinated	137 (51.10)	98 (51.30)			
Are you considering additional vaccinations for your child (e.g., against flu, HPV)?					
Yes	129 (48.10)	103 (53.90)	1.26	0.839–3.368	0.13
No	139 (51.90)	88 (46.10)			
Do you plan a vaccinate your child in the future according to the vaccination schedule?					
Yes	230 (85.80)	167 (87.40)	1.15	0.855–1.862	0.36
No	38 (14.20)	24 (12.60)			
Mistrust_VAX1					
Low Mistrust	217 (81.00)	153 (80.10)	1.06	0.644–2.085	0.45
High Mistrust	51 (19.00)	38 (19.90)			
Worry_VAX2					
Minor Worry	87 (32.50)	83 (43.50)	1.60	0.641–1.730	0.11
Severe Worry	181 (67.50)	108 (56.50)			
Concerns_VAX3					
Mild Concerns	203 (75.70)	148 (77.50)	0.91	1.069–2.389	0.71
Severe Concerns	65 (24.30)	43 (22.50)			
Preferences					
Weak Preferences	211 (78.70)	147 (77.00)	1.11	0.569–1.439	0.37
Strong Preferences	57 (21.30)	44 (23.00)			

**Table 10:** Awareness and perceptions of vaccination information.

Answers to the Questionnaire Questions	N (%) N = 459
Have there been any vaccination information campaigns organized at your child’s school?	
Yes	149 (32.46)
No	140 (30.50)
Undecided	170 (37.04)
In your opinion, is vaccination-related information sufficiently accessible?	
Yes	206 (44.88)
No	156 (33.99)
No opinion	97 (21.13)
Do you believe schools should be more involved in vaccination education?	
Yes	231 (50.33)
No	70 (15.25)
Undecided	158 (34.42)
Are you aware of individuals in your social circle who choose not to vaccinate their children?	
Yes	281 (61.22)
No	139 (30.28)
Unknow	39 (8.50)

## Data Availability

All data in the study are available from the corresponding author upon request.

## Supplementary Materials

The following supporting information can be downloaded at: https://www.mdpi.com/article/10.3390/vaccines13080869/s1.
