# Supplementary material for: Knowledge and Attitudes of Parents of School-Aged Children Regarding Vaccinations, and an Analysis of Measles and Pertussis Vaccination Coverage Using the Example of the City of Radomsko in Central Poland
Source: Vaccines (Basel). 2025 Aug 16;13(8):869. doi: 10.3390/vaccines13080869 (PMC12389980; doi:10.3390/vaccines13080869)
Supplement: Supplementary file 1 [file vaccines-13-00869-s001.zip › Questionnaire_Nowicki_UMedPoland.pdf]

UNIWERSYTET MEDYCZNY W ŁODZI

Dear Sir/Madam,

I am a 3rd-year Public Health student at the Medical University of Łódź. As part of my thesis project conducted at the **Department of Nutrition and Epidemiology, Medical University of Łódź**, I am researching:

*" **Assessment of the knowledge of parents of school-age children about vaccinations and analysis of the vaccination status against measles and pertussis in the city of Radomsko.”***

The thesis supervisor is **Anna Garus-Pakowska, MD, PhD, Prof. of UMED**.

The study aims to:

1. Assess parental knowledge about vaccinations.
2. Analyze measles and pertussis vaccination coverage among school-aged children (grades 1–3, birth years 2014–2019) in Radomsko.
3. Identify factors influencing parental vaccination decisions.

Participation is voluntary and anonymous. Data will not be shared with external entities. The **Medical University of Łódź** is the data controller.

Thank you for your time.
Paweł Nowicki

---

1. I have read the study objectives and voluntarily consent to complete this anonymous questionnaire

a) Yes
b) No

Part 1: Demographic Information

1. Age: ___________
2. Gender:
   a) Female
   b) Male
   c) Other
   d) Prefer not to answer
3. Education level:
   a) Primary
   b) Secondary
   c) Bachelor’s degree

Questionnaire Umed Łódź

d) Master's degree
e) PhD or higher

4. Self-assessed financial status:
a) Low
b) Satisfactory
c) High
5. Employment status:
a) Employed
b) Unemployed
c) Student
6. Number of children in grades 1–3 (birth years 2014–2019):
a) 1
b) 2
c) 3
d) 4 or more

Part 2: Vaccination Knowledge

7. Are you familiar with the concept of *immunization*?
a) Yes
b) No
c) Unsure
8. Do you know Poland's mandatory vaccination schedule?
a) Yes
b) No
c) Aware but unfamiliar with details
9. Which diseases are covered by *mandatory* vaccinations in Poland? (Select all that apply):
a) Measles
b) Pertussis
c) Polio
d) Varicella
e) Diphtheria
f) Tetanus
g) Hepatitis B
h) Hepatitis A
i) Other (specify): ___________
j) Don't know
10. Which vaccines are *recommended* (non-mandatory) in Poland? (Select all that apply):
a) Influenza
b) Hepatitis A
c) Tick-borne encephalitis (TBE)
d) Varicella
e) HPV
f) Rotavirus
g) Pneumococcal
h) Meningococcal

Questionnaire Umed Łódź

i) Other (specify): ___________
j) Don't know

11. What is the *primary benefit* of vaccination? (Select one):
    a) Protection against infectious diseases
    b) Reduced epidemic risk
    c) Protection of unvaccinated individuals (herd immunity)
    d) Reduced disease severity
    e) No perceived benefits
    f) Other (specify): ___________
12. Consequences of *not vaccinating* a child? (Select all that apply):
    a) Increased disease susceptibility
    b) Higher complication risk
    c) Risk to others
    d) Potential exclusion from school/daycare
    e) No perceived negative consequences
    f) Other (specify): ___________
13. Most common *adverse effects* of vaccines? (Select all that apply):
    a) Injection-site pain
    b) Fever
    c) Rash
    d) Fatigue
    e) Prolonged crying
    f) Other (specify): ___________
14. Have you heard of the *MMR vaccine* (measles, mumps, rubella)?
    a) Yes
    b) No
15. Do you understand why vaccinations are important for *public health*?
    a) Yes
    b) No
    c) Unsure
16. Are you familiar with the term *herd immunity*?
    a) Yes
    b) No
    c) Aware but unfamiliar with details
17. Which disease has been *globally eradicated* through vaccination?
    a) Tetanus
    b) Measles
    c) Rubella
    d) Smallpox
    e) HPV
    f) Polio
    g) Plague
    h) None

Part 3: Vaccination Practices (for children in grades 1–3, born 2014–2019)

18. Has your child received *all mandatory vaccinations*?
    a) Yes

Questionnaire Umed Łódź

b) No
c) Unsure

19. If not vaccinated, state the reason(s):
    a) Vaccine safety concerns
    b) Lack of knowledge
    c) Child's health issues
    d) Religious/philosophical beliefs
    e) Lack of healthcare provider support
    f) Vaccine access barriers
    g) Previous adverse reactions
    h) Other (specify): ___________
20. Has your child been vaccinated against *measles*?
    a) Yes, all doses
    b) Yes, one dose
    c) Yes (unsure of doses)
    d) No
    e) Unsure
21. Has your child been vaccinated against *pertussis*?
    a) Yes, all doses
    b) Yes, one dose
    c) Yes (unsure of doses)
    d) No
    e) Unsure
22. Did your child experience *adverse reactions* post-vaccination?
    a) After measles vaccine
    b) After pertussis vaccine
    c) After both
    d) No
    e) Unsure
23. If adverse reactions occurred, were they *reported to a physician*?
    a) Yes
    b) No
    c) Not applicable
24. Do you plan to vaccinate your child per the *national schedule*?
    a) Yes
    b) No
    c) Undecided
25. Would you consider *optional vaccines* (e.g., influenza, HPV)?
    a) Yes
    b) No
    c) Undecided
26. What influences your vaccination decisions? (Select all that apply):
    a) Physician's recommendation
    b) Media (TV, radio)
    c) Internet sources
    d) Family/friends' opinions
    e) Personal beliefs
    f) Other (specify): ___________
27. Potential *complications of measles*? (Select all that apply):
    a) Pneumonia
    b) Encephalitis

Questionnaire Umed Łódź

c) Skin rash
d) Vision impairment
e) Don’t know

28. Potential *complications of pertussis*? (Select all that apply):
a) Pneumonia
b) Breathing difficulties
c) Severe coughing fits
d) Brain damage
e) Don’t know

Part 4: Information Access and Attitudes

29. Has your child’s school organized *vaccination awareness campaigns*?
a) Yes
b) No
c) Don’t know

30. Is vaccination information *sufficiently accessible*?
a) Yes
b) No
c) No opinion

31. Should schools *increase involvement* in vaccination education?
a) Yes
b) No
c) No opinion

32. Do you know anyone who *does not vaccinate* their children?
a) Yes
b) No
c) Unsure

Part 5: Vaccination Attitudes Scale

Please mark the answers that best reflect your beliefs or feelings. There are no right or wrong answers here.

1. **I feel safe after** being vaccinated. **(-)**

Strongly Disagree					Strongly Agree
**1**	**2**	**3**	**4**	**5**	**6**

2. I can rely on vaccines to stop serious infectious diseases. (-)

Strongly Disagree					Strongly Agree
**1**	**2**	**3**	**4**	**5**	**6**

Questionnaire Umed Łódź

3. I feel protected after getting vaccinated. (-)

Strongly Disagree					Strongly Agree
**1**	**2**	**3**	**4**	**5**	**6**

4. Although most vaccines appear to be safe, there may be problems that we haven't yet discovered.

Strongly Disagree					Strongly Agree
**1**	**2**	**3**	**4**	**5**	**6**

5. Vaccines can cause unforeseen problems in children.

Strongly Disagree					Strongly Agree
**1**	**2**	**3**	**4**	**5**	**6**

6. I worry about the unknown effects of vaccines in the future.

Strongly Disagree					Strongly Agree
**1**	**2**	**3**	**4**	**5**	**6**

7. Vaccines make a lot of money for pharmaceutical companies, but don't do much for regular people.

Strongly Disagree					Strongly Agree
**1**	**2**	**3**	**4**	**5**	**6**

8. Authorities promote vaccination for financial gain, not for people's health.

Strongly Disagree					Strongly Agree
**1**	**2**	**3**	**4**	**5**	**6**

9. Vaccination programs are a big con.

Strongly Disagree					Strongly Agree
**1**	**2**	**3**	**4**	**5**	**6**

10. Natural immunity lasts longer than a vaccination.

Strongly Disagree					Strongly Agree

Questionnaire Umed Łódź

**1**	**2**	**3**	**4**	**5**	**6**

11. Natural exposure to viruses and germs gives the safest protection.

Strongly Disagree					Strongly Agree
**1**	**2**	**3**	**4**	**5**	**6**

12. Being exposed to diseases naturally is safer for the immune system than being exposed through vaccination.

Strongly Disagree					Strongly Agree
**1**	**2**	**3**	**4**	**5**	**6**

After reverse-coding, scale and subscale scores are created by averaging the relevant items (1, 2, 3 = mistrust of vaccine benefit; 4, 5, 6 =
worries over unforeseen future effects; 7, 8, 9 = concerns about commercial profiteering; 10, 11, 12 = preference for natural immunity).

Questionnaire Umed Łódź
