# Supplementary figures and images for: Knowledge and Attitudes of Parents of School-Aged Children Regarding Vaccinations, and an Analysis of Measles and Pertussis Vaccination Coverage Using the Example of the City of Radomsko in Central Poland
Source: Vaccines (Basel). 2025 Aug 16;13(8):869. doi: 10.3390/vaccines13080869 (PMC12389980; doi:10.3390/vaccines13080869)

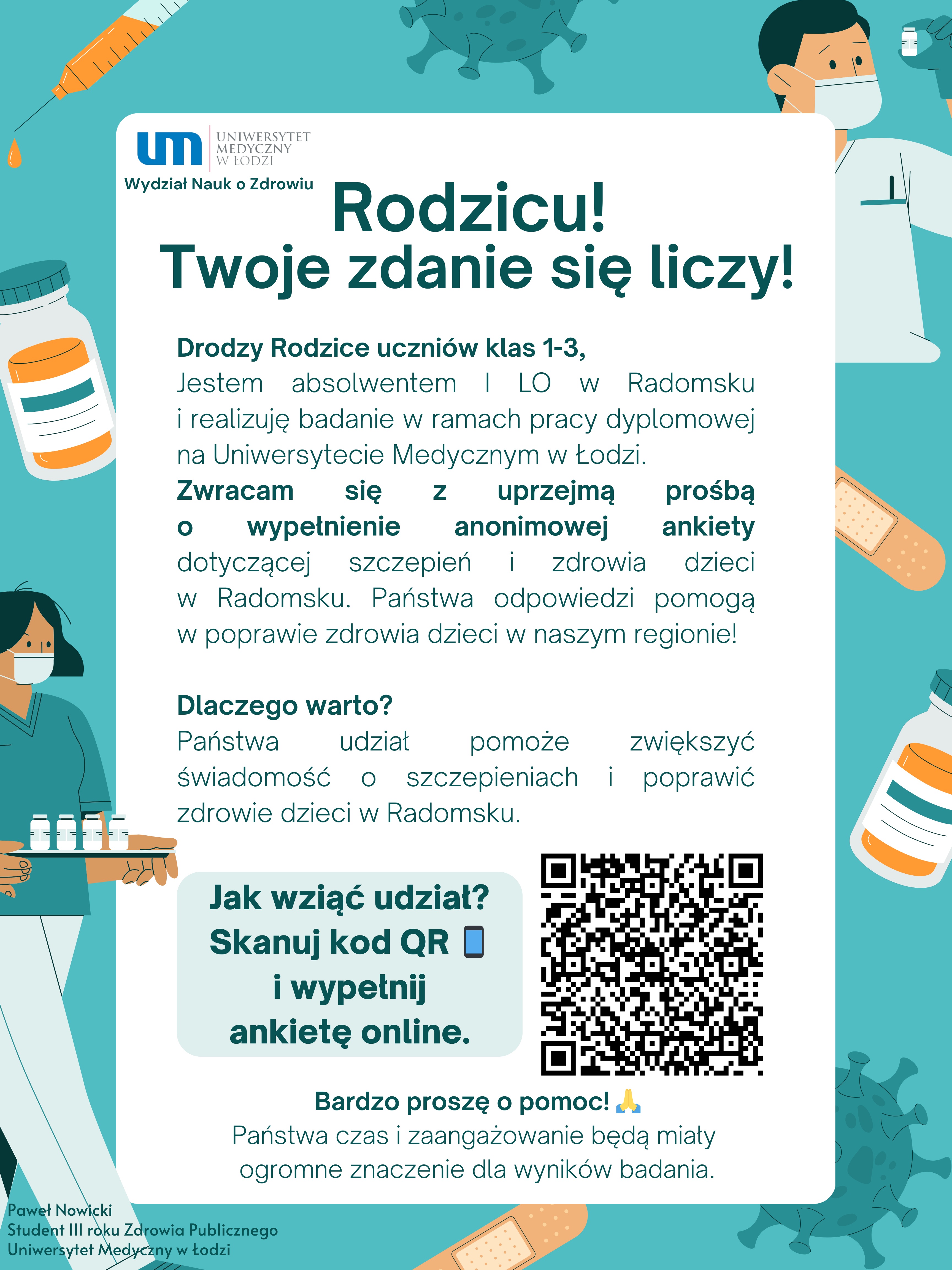

Supplement: Supplementary file 1 [file vaccines-13-00869-s001.zip › PLAKAT_NOWICKI.jpg]
